# Physical Growth Charts from Birth to Six Years of Age in Japanese Twins

**DOI:** 10.2188/jea.14.151

**Published:** 2005-03-18

**Authors:** Syuichi Ooki, Yoshie Yokoyama

**Affiliations:** 1 Department of Health Science, Ishikawa Prefectural Nursing University.; 2 Faculty of Health Sciences, Okayama University Medical School.

**Keywords:** Twins, Growth, Weight Gain, Japan

## Abstract

BACKGROUND: The purpose of this study was to analyze the characteristics of the physical growth of twins in childhood and to present growth charts of Japanese twins.

METHODS: The subjects consisted of 2029 pairs of normally developed Japanese twins. Growth data were obtained by mailed or hand-distributed questionnaires. Factors that affect body weight and height/length at selected ages were analyzed by stepwise regression analysis. Selected percentiles o body weight, height/length, and body mass index were calculated according to sex, and growth curves were drawn using a spline function. The size deficit of the twins compared to the standards for the general population of Japan was calculated.

RESULTS: Gestational age, parity, zygosity, and birth order affected physical growth in varying degrees, although the overall effects themselves were small and mostly disappeared by one year of age. Growth charts of the twins present growth at selected percentiles from birth to 6 years of age according to sex. The size deficit of the twins was largest at birth: more than 20% for weight and approximately 6% for length compared to the 50th percentile of the standard for the general population of Japan. These deficits decreased rapidly in the first 6 to 12 months, and were found to be as low as 0-2% at 4 to 6 years of age.

CONCLUSION: Growth charts specifically for twins are needed, at least for the first 1 to 3 years of age but not beyond the age of 6 years.

In Japan, as in other developed countries, the rate of multiple births has been increasing since 1975. The higher twinning rates in Japan since 1987 have been attributed to both the higher proportion of mothers treated with ovulation-inducing hormones and to the increasing use of in vitro fertilization.^1^ Currently about 1% of all births are multiples.^2^ Therefore, there is an increasing need to provide appropriate information to parents and nursing staff on the biological characteristics of twins, such as their intrauterine growth, physical growth after birth, and motor and language development.^3^ However, little information is available, in part because the lack of a population-based twin registry makes it difficult to collect growth data on twins, especially after birth. There have been many studies on the physical growth of twins in childhood in Western countries,^4^^-^^10^ including Wilson’s detailed summary of the features of twin growth.^11^

It is well known that the growth patterns of twins in utero and in childhood are very different from those of singletons.^3^^,^^12^^-^^14^ Because there are no growth standards for twins in Japan, the physical growth of twins in childhood must be evaluated using the standards for the general population^15^ — that is, standards for singletons. As a result, many twins are regarded as having poor growth, especially when they are very young, and this causes both the twins and their parents much embarrassment and concern. In general in Japan, pediatricians and public health nurses, the professional advisors for the growth of children, do not have adequate information to answer questions that the parents of twins ask about their children’s growth. Moreover, many factors that affect body size after birth, such as the perinatal medical system, body size at birth, and the body size of the mother, differ significantly between Japan and Western countries. To resolve these shortcomings in appropriate data, it is essential to obtain objective growth data based on a large sample of Japanese twins.

There are two dimensions to estimating the growth of twins: comparing individual twins versus singletons,^6^^,^^9^ and comparing the similarity of the twins within a pair by zygosity.^5^^,^^8^^,^^10^ The present study deals with twins as individuals. The purpose of this study is to analyze the characteristics of twins’ physical growth from birth to 6 years of age and to present growth charts of normally developed Japanese twins in comparison with those of the general population. This comparison between twin and singleton standards may also be useful in growth studies of Caucasians.

## METHODS

### Subjects

In Japan only two main sources of data about multiple births have been established, and the gap between them is wide. First, vital statistics may be available without access to information on specific individuals. Second, data from hospitals have been used in the field of obstetrics, mainly in managing high-risk pregnancies. Although this method is relatively easy to use, it may allow large selection biases to occur.^3^ To cover the gap between vital statistics and hospital data, the authors have been constructing a twin database that would contribute to overall health care for families with multiples. The database is larger and less biased than hospital data and its information is more detailed than the vital statistics, especially as to zygosity. In addition, the authors made much of afterbirth data, which neither vital statistics nor hospital data adequately supply. The basic characteristics of this database are described in detail elsewhere (Ooki et al: Construction of Japanese database on child twins and their families; submitted to the journal *Twin Research*). The present data were drawn from this database and were obtained by questionnaire.

The subjects of this study were 2029 mothers and their 4058 twin children, who were recruited from among two different groups. The first group of subjects consisted of 937 mothers and their 1874 twins who belonged to various associations for parents of multiples throughout Japan (maternal associations group). The second group of subjects consisted of 1092 mothers and their 2184 twins living in the Tokyo metropolitan area; all of the twins in this group had applied to (though not necessarily enrolled in) the secondary education school attached to the faculty of education of the University of Tokyo between 1982 and 2003 (school applicants group). In the maternal associations group the twins were born between 1986 and 2002, and in the school applicants group the twins were born between 1968 and 1991. At the time data were collected, in 2002-2003, the twins in the maternal associations group ranged in age from 0 to 15 years (mean 6.0 years, standard deviation 3.8 years, median age 5.0 years), and the twins in the school applicants group were all 11 or 12 years old.

### Data collection

Data were collected through mailed or hand-delivered questionnaires, which had nearly the same format between the two groups. The questions asked about family structure; obstetrical findings on the mothers; the twins’ physical growth, zygosity, motor, language, and mental development; the twins’ and parents’ medical histories; and any behavioral problems the twins had had. In the school applicants group, one parent of each twin, usually the mother, participated in a medical interview by two or three interviewers, including, from 1988 on, one of the present authors (Ooki), in which their responses to the questionnaire were checked carefully.

Detailed obstetrical records on the mothers in the school applicants group were also obtained from the “Maternal and Child Health Handbook”, which the Ministry of Health, Labor and Welfare of Japan provide to all pregnant women. No complete information on chorionisity was obtained.

In Japan, the health examination system after birth differs according to life stage. This has made it difficult to obtain continuous growth data on children before and after 6 years of age. Until 6 years of age, children receive a health examination administered by the Ministry of Health, Labor and Welfare based on age, which is counted as real weeks, months, or years after birth. The “Maternal and Child Health Handbook” presents the growth standards of weight and height/recumbent length and developmental milestones every 10 years, e.g., 1980, 1990,^15^ and 2000. The growth data of children based on mass examinations are usually recorded in this Handbook. The authors of the present study advised the mothers to refer to these records when completing the questionnaire. Growth data were assigned to the appropriate age groups on the basis of days since birth, which was calculated as the date at mass examination minus the child’s birthday. For example, if the subject’s age was 73 days, his or her data were assigned to the 2- to 3-month-old group. The data from the Handbook were accepted without modification; therefore, the method and accuracy of those measurements could not be ascertained.

Although this method seemed to be an effective way to collect large amounts of growth data on twins after birth, it did not produce perfect longitudinal data, and there were certain differences in the number of subjects in each age group.

### Zygosity classification

The zygosity of the twins was determined primarily by a questionnaire^16^ that was used widely in Japan and that, in the present study, was completed by the mothers in both groups. The zygosity types were monozygotic, unclassified, and dizygotic, according to the similarity score, which was calculated by using five questions regarding the physical similarity and confusion of identity between the twins. The accuracy in classifying their twins was nearly 98%, although about 10% of pairs were unclassified.^16^ The accuracy was a trade-off according to the percentage of unclassified pairs.

For the school applicants group, zygosity was diagnosed also by the use of many genetic markers for those twin pairs who were actually admitted to the school.^3^ The twins in the present study were classified as 1159 monozygotic, consisting of 557 male-male and 602 female-female pairs; 675 dizygotic, consisting of 170 male-male, 174 female-female, 163 male-female, and 168 female-male pairs; and 195 unclassified, consisting of 106 male-male and 89 female-female pairs. As zygosity testing is very rare in Japan, this is the largest sample to date for a study on growth data of twins in Japan that includes both correct age and specified zygosity.

### Preliminary analyses and data combination

**Figure 1.**
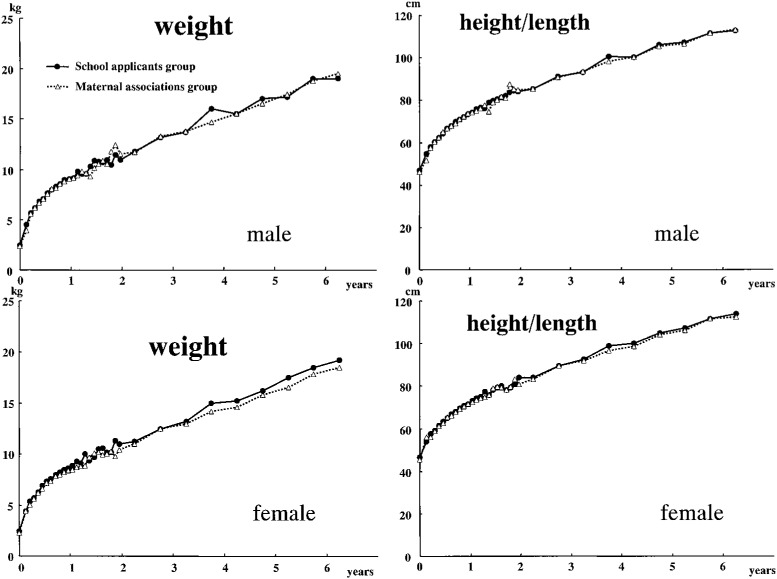
The 50th percentiles of body weight and height/length from birth to six years of age (raw data).

Before the data of the two groups were combined, weight and height in both groups were examined in detail. The results are described elsewhere (Ooki et al. *Twin Research*, mentioned above). Birth complications of mothers or twins, such as placenta previa, placental abruption, coiling of the umbilical cord, neonatal asphyxia, growth-discordant twins, and twin-to-twin transfusion syndrome, were observed to varying degrees. None of these were grounds for exclusion from the study. In general, it was very difficult to set clear and consistent inclusion/exclusion criteria, as more than 10% of the present subjects had at least one of the complications mentioned above. Moreover, no subjects showed apparent retardation of physical growth at the time of data collection. The preliminary analyses are summarized as follows. (1) The twins in the maternal associations group had one-week shorter gestations than the twins in the school applicants group. (2) Body size parameters at birth, namely weight, length, chest circumference, and head circumference were slightly smaller in the maternal associations group. Nevertheless, both birth weight itself according to gestational weeks and the percentage difference in relative birth weight within pairs were nearly the same compared to the birth weight norms of the general twin population in Japan.^17^ (3) The two groups were very similar to each other in body weight and height from birth through 6 years of age, as shown in Figure 1. For example, the range of body weight difference between the groups (the school applicants group minus the maternal associations group) at the 50th percentile of the raw data was from -1.30 kg to 1.30 kg for males and -0.37 kg to 1.50 kg for females. These findings suggested that the groups were not very different, at least in their physical development. Moreover, in both groups the data seemed to reflect normal physical development after birth. All data samples were used, irrespective of the data source, in order to maintain the sample size, stabilize the monthly measurement values, and not overestimate monthly measurement values from birth to one year of age.

### Statistical analysis

Birth complications were not grounds for exclusion from the following analysis. Usually, the growth standards of singletons *in utero*, determined by body size at birth according to the gestational weeks, are presented according to sex and/or parity (primiparity or multiparity), whereas after birth the standards are presented according to sex alone. In the case of twins, at least two additional factors that are not relevant to singletons might also be considered: the birth order of the twins and their zygosity (monozygotic or dizygotic).

First, the factors that affected the body weight and height/length of twins at selected ages were confirmed by stepwise regression analysis, with a threshold significance level of 0.05. The variables considered were sex, birth order of the twins, gestational weeks, maternal and paternal age upon the birth of the twins, parity, zygosity, presentation, the birth year of the twins, and the group to which the subjects belonged. For qualitative variables, the following codes were used: sex, female 0, male 1; birth order, first-born 1, second-born 0; zygosity, monozygotic 0, dizygotic 1; presentation, non-vertex 0, vertex 1; group, maternal associations group 1, school applicants group 0. The numbers of subjects on whom data were missing as to parity, presentation, zygosity, gestational age, maternal age, and paternal age were 4, 446, 390, 52, 36, and 8, respectively. There were no missing data for sex, birth order, or birth year of the twins.

Growth charts were developed from the results of the regression analysis. The selected percentiles (3rd, 10th, 25th, 50th, 75th, 90th, and 97th) of body weight (kg), height/length (cm), and body mass index (kg/m^2^) at each age were calculated, and growth curves were drawn by means of a spline function. Body mass index (BMI) is mathematically equal to the Kaup index, which has been widely used in Japan as an index for the physique or obesity of a child younger than 6 years. Though several problems were pointed out regarding BMI as an indicator of child obesity, this index has become a world standard.^18^ Table 1 shows the number of subjects using data analysis according to age and group.

**Table 1.** Number of subjects according to sex, age, and group.

		weight				height/length			
	
		male		female		male		female	
			
		M group	S group	M group	S group	M group	S group	M group	S group
	at birth	966	1022	899	1154	944	957	868	1092
0 year	2-3 months	95	152	68	149	91	138	62	136
	3-4	481	514	469	554	456	490	448	534
	4-5	357	199	315	275	351	193	312	270
	5-6	171	202	157	201	164	191	152	193
	6-7	420	374	405	425	410	367	389	419
	7-8	209	98	216	103	201	97	211	98
	8-9	159	95	141	108	157	94	138	98
	9-10	337	263	320	304	327	259	312	302
	10-11	242	132	263	137	238	129	262	132
	11-12	178	100	145	88	174	98	142	88
1 year	0-1	318	288	358	358	314	285	348	352
	1-2	94	78	93	58	90	76	91	58
	6-7	178	97	135	117	178	95	134	116
	7-8	94	61	75	79	95	61	75	78
2 years	0-6	335	236	344	265	328	234	333	259
	6-12	91	69	67	56	85	67	68	56
3 years	0-6	545	647	528	719	540	648	527	715
	6-12	155	65	147	65	153	65	147	68
4 years	0-6	201	239	203	257	198	239	206	259
	6-12	90	120	89	113	88	120	91	115
5 years	0-6	192	244	190	245	192	244	190	247
	6-12	72	103	54	110	70	103	54	110
6 years	0-6	171	323	184	364	171	324	181	371

M group: Maternal associations group.

S group: School applicants group

The size deficit in twins was calculated as the percentage difference between the value of the general population and that of the twins divided by the value of the general population. Size deficits were calculated using the 50th percentile values of the growth standards presented by the Ministry of Health and Welfare (now reorganized as the Ministry of Health, Labor and Welfare) in 1990.^15^ All data were analyzed using SAS^®^ for Windows.^19^ The growth curves were smoothed by the PROC TRANSREG procedure, with the ‘pspline’ model specified.

### Ethical issues

The mothers in the maternal associations group all cooperated voluntarily in this research, mainly through their associations. Informed consent concerning the statistical analysis of the data in the school applicants group was obtained from each twin and his or her parents in writing as part of the application process.

## RESULTS

The results of the stepwise regression analysis are shown in Table 2. Gestational weeks, sex, parity, zygosity, and birth order significantly affected both birth weight and birth length. None of the other variables — that is, the maternal and paternal age upon the birth of the twins, presentation, the birth year of the twins, or the group — met the 0.05 significance level for entry into the model. Lasting effects of gestational weeks and sex were found with statistical significance up to at least 3 years of age. The contributions of other factors were negligible and mostly disappeared by 6 to 12 months of age.

**Table 2.** The results of stepwise regression analysis for weight and height/length.

	weight										height/length									
	
	gestational age		parity		sex		birth order		zygosity		gestational age		parity		sex		birth order		zygosity	
									
	R^2^ (%)	p	R^2^ (%)	p	R^2^ (%)	p	R^2^ (%)	p	R^2^ (%)	p	R^2^ (%)	p	R^2^ (%)	p	R^2^ (%)	p	R^2^ (%)	p	R^2^ (%)	p
birth	39.78	**	1.91	**	0.88	**	0.76	**	0.08	*	36.56	**	1.09	**	1.33	**	0.25	**	0.11	*
3 months	12.28	**	-	n.s.	7.34	**	0.47	**	-	n.s.	18.03	**	0.18	*	6.91	**	0.16	*	-	n.s.
6 months	3.44	**	0.63	**	8.21	**	0.53	**	-	n.s.	6.24	**	0.44	*	10.46	**	-	n.s.	-	n.s.
9 months	4.36	**	0.32	*	8.18	**	0.43	*	-	n.s.	6.30	**	-	n.s.	8.03	**	-	n.s.	-	n.s.
1 year	3.98	**	-	n.s.	8.66	**	-	n.s.	-	n.s.	6.22	**	-	n.s.	6.07	**	-	n.s.	-	n.s.
2 years	1.17	**	-	n.s.	8.32	**	-	n.s.	-	n.s.	1.61	**	-	n.s.	5.99	**	-	n.s.	-	n.s.
3 years	0.69	**	-	n.s.	4.16	**	-	n.s.	-	n.s.	0.84	**	-	n.s.	2.49	**	-	n.s.	-	n.s.

*: p<0.05, **: p<0.01.

None of the other variables (maternal and paternal age upon the birth of the twins, presentation, the birth year of the twins, or subject group) met the 0.05 significance level for entry into the model.

R^2^: coefficient of determination

The coefficient of determination (R^2^) of gestational weeks rapidly decreased with age, while the effect of sex did not. Physical growth from birth to 3 years of age was calculated according to sex and gestational weeks. The results are shown in Tables 3 and 4. The gestational weeks were divided into two groups: 25-36 weeks (preterm delivery) and 37-41 weeks (term delivery). The difference in weight or height/length between the preterm and term groups was calculated as the percentage difference between the value of the term group and that of the preterm group divided by the value of the term group. Between the term and preterm groups, the difference in weight was nearly 20% at birth, rapidly decreased until 3 months, and was within 5% after 6 months. As to height/length, the difference was nearly 6% at birth and gradually decreased after birth. Since there were many detailed standards for birth weight and length in twins according to gestational weeks^3^, growth charts after birth were calculated separately for males and females without taking gestational periods into consideration, following the system used to calculate standards for singletons.^20^

**Table 3.** The 50th percentiles of weight from birth to 3 years of age according to gestational weeks (preterm vs term delivery).

	weight									

	male					female				
	
	25-36 weeks (preterm)		37-41 weeks (term)		difference	25-36 weeks (preterm)		37-41 weeks (term)		difference
					
	n	kg	n	kg	%	n	kg	n	kg	%
birth	677	2.13	1276	2.61	18.4	656	2.04	1348	2.54	19.7
3 months	344	5.82	636	6.26	7.0	332	5.31	662	5.84	9.1
6 months	245	7.52	540	7.79	3.5	262	7.00	558	7.37	5.0
9 months	199	8.42	397	8.74	3.7	205	7.96	405	8.17	2.6
1 year	208	9.09	386	9.33	2.6	229	8.48	473	8.80	3.6
2 years	209	11.80	355	11.86	0.5	206	10.93	394	11.26	2.9
3 years	400	13.70	771	13.83	0.9	383	13.05	836	13.33	2.1

Difference=(T-P)/T × 100. P: weight of preterm delivery, T: weight of term delivery.

**Table 4.** The 50th percentiles of height/length from birth to 3 years of age according to gestational weeks (preterm vs term delivery).

	height/length									

	male					female				
	
	25-36 weeks (preterm)		37-41 weeks (term)		difference	25-36 weeks (preterm)		37-41 weeks (term)		difference
					
	n	cm	n	cm	%	n	cm	n	cm	%
birth	619	44.6	1247	47.4	5.9	606	44.0	1313	46.8	6.0
3 months	314	58.7	618	60.6	3.1	318	56.9	637	59.2	3.9
6 months	235	65.9	533	67.0	1.6	249	64.1	549	65.5	2.1
9 months	193	70.0	389	71.1	1.5	201	68.5	399	69.5	1.4
1 year	204	73.4	383	74.3	1.2	225	71.9	461	73.1	1.6
2 years	206	84.9	349	85.5	0.7	200	83.2	383	84.0	1.0
3 years	400	93.2	767	93.6	0.4	383	92.0	831	92.6	0.6

Difference=(T-P)/T × 100. P: height/length of preterm delivery, T: height/length of term delivery.

**Figure 2.**
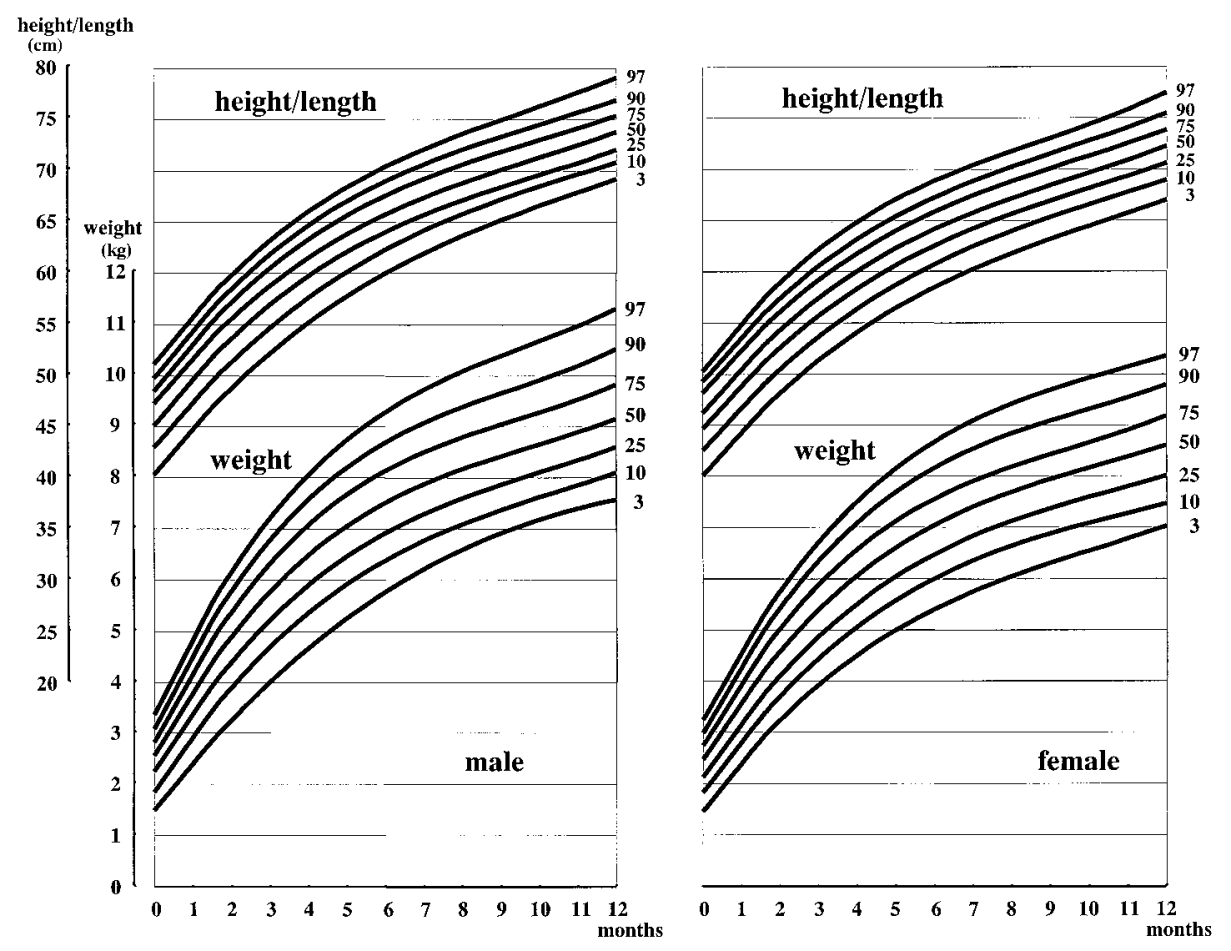
Body weight and height/length of twins by age percentiles from birth to 1 year of age.

**Figure 3.**
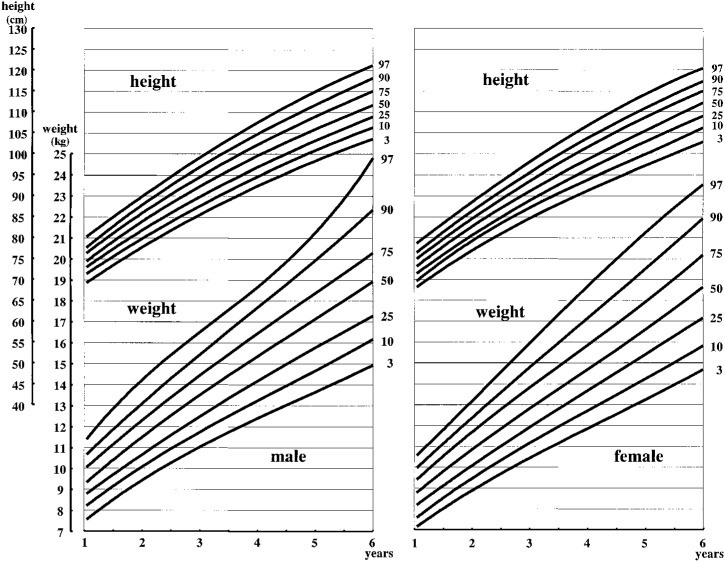
Body weight and height of twins by age percentiles from 1 to 6 years of age.

**Figure 4.**
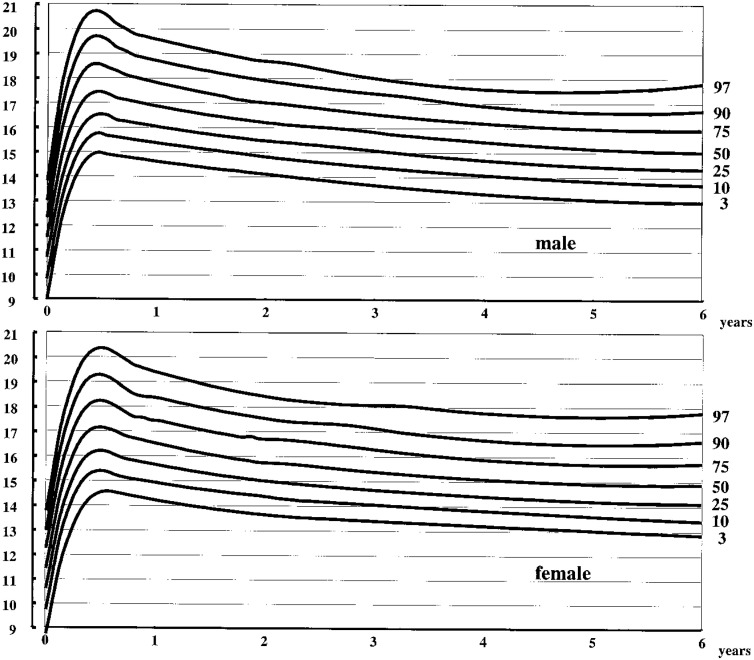
Body mass index of twins by age percentiles from birth to 6 years of age.

The 3rd, 10th, 25th, 50th, 75th, 90th, and 97th percentile curves of weight and height/length from birth to 1 year of age are presented in Figure 2, and those from 1 to 6 years of age are presented in Figure 3. The 3rd, 10th, 25th, 50th, 75th, 90th, and 97th percentile curves of BMI from birth to 6 years of age are presented in Figure 4.

**Figure 5.**
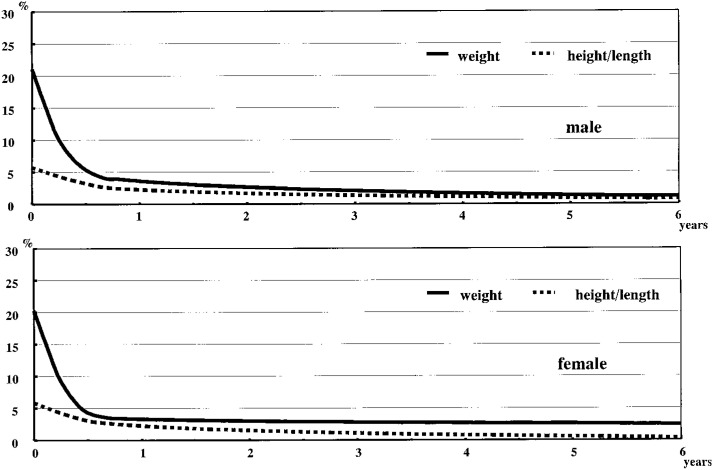
Size deficit of twins as to the 50th percentiles compared with general population from birth to 6 years of age.

The size deficits of twins in weight and height/length from birth to 6 years of age are presented in Figure 5. The weight deficit of twins in the 50th percentile was more than 20% at birth relative to the growth standards of the general population. However, the deficit decreased rapidly, to approximately 5% within the first 6 months, and diminished thereafter. The length deficit of the twins at the 50th percentile was approximately 6% at birth and gradually decreased, reaching about 3% by 7 to 8 months of age. It then decreased slightly thereafter.

## DISCUSSION

There are no growth charts for twin children in Japan despite the rapid increase in multiple births and the increasing need to provide appropriate information to parents and nursing staff. Most studies reported so far regarding the physical growth of twins after birth in Japan had very small samples and therefore only roughly classified age after birth. The present data are the largest twin sample in Japan to provide accurate age after birth and information on zygosity.

The effects of parity, birth order, and zygosity in twins on their birth weight and length, irrespective of gestational age, have been extensively reported.^21^^-^^23^ Nevertheless, in the present study those effects were small and mostly disappeared at an early age, as shown in Table 2. Although lasting effects of gestational periods were observed statistically, we found, as can be seen in Tables 3 and 4, that these effects decreased constantly after birth and the impacts themselves were not necessarily large except at birth. The effects of gestational age on birth weight or length have been analyzed many times in greater detail.^3^ Moreover, the growth standards of the general population do not correct the effects of gestational periods to reflect the actual conditions of physical growth.^20^ To estimate the difference between the general population and twins, the present data should be treated in the same way. Therefore, in this study the growth charts for the twins were differentiated only by sex.

Compared with the general population, the twins’ size deficit was greatest at birth but decreased dramatically in the first 6 to 12 months, reaching as little as approximately 2%, around 1 kg for weight and 1 cm for height, by 6 years of age. This finding was consistent with the data reported by Wilson.^11^ It was very difficult to determine when or whether twins fully catch up to singletons, as the present study did not use a complete birth cohort containing singletons. Since it is found here that most of the size deficit of twins compared to the general population is gone by 6 years of age, there is no practical necessity for specific growth charts for twins after that age.

The limitations of the present study were as follows. First, the data were obtained from retrospective records, and the methods of measurement were not necessarily consistent. Nevertheless, the data on physical growth did not reveal a large difference between the maternal associations group and the school applicants group, as shown in Figure 1. This suggested the absence of any fatal methodological problems in data collection.

The second limitation was that these data were semi-longitudinal rather than cross-sectional. Specifically, data on the same individual were used according to the recorded times. For example, some twins provided almost longitudinal data. On the other hand, others provided only birth weight. Additionally, the number of subjects in each age group varied considerably, as shown in Table 1. Consequently, the range of measurements in each age group becomes smaller than it would be in complete cross-sectional data, such as the standards for the general population.^15^ The clinical use of 3rd percentiles and 97th percentiles of this growth charts as indicators of growth retardation is not necessarily appropriate.

Third, as all data were derived from normally developed twins, the norm for the early days after birth may have been overestimated. It is difficult to specify the direct effects of these selection biases. However, compared with the hospital data, which usually include many high-risk subjects, the present data seem to reflect more closely the physical growth features of the general twin population.^3^

Fourth, as the birth years of the present subjects ranged from 1968 to 2002, a period of 35 years, yearly trends in physical growth may also be considered. Fortunately, no birth-year effects were observed in the present study, as mentioned in the results of the stepwise regression analysis. According to the physical growth standards of the general population, birth weight in Japan began to decline after 1975, with the result that body size is smaller until several years after birth. The reduction in infant body size has been attributed to other factors, too, such as lifestyle changes. Although the growth standards presented by the Ministry of Health and Welfare in 1990^15^ were used in the present study, the difference between twins and singletons was smaller according to the growth standards presented in 2000. Given this trend, it is difficult to achieve a meaningful comparison of twin growth and singleton growth. Body weight and height after school entrance in both groups of subjects were not analyzed, because the present data did not accommodate the much larger secular trends at this life stage.

Fifth, physiological body weight loss after birth was not assessed because of a lack of appropriate data. Such weight loss was presented as to the growth standards in the general population.^15^

Finally, the merits and demerits of combining the data of the two subject groups should be discussed. One reason why we combined the data was that the groups were of similar size. Preliminary analysis showed that the body size parameters of the twins at birth were slightly lower in the maternal associations group, whereas physical growth after birth was nearly identical between the groups. Moreover, it is not always the case that growth charts are developed based on only one group of subjects. Usually, complicated methods of weighting measurements and modifying those measurements according to the number of subjects and so on are part of the development of growth charts; see, for example, “2000 CDC Growth Charts: United States”.^24^ For the present data, no special corrections were performed. Some unexpected selection biases or confounding may have occurred. These effects would be estimated by comparing the data to less biased, more representative, and nationwide data on twins.

Although selection bias should be quite low in a population-based study, it is almost impossible to conduct this type of twin study in Japan at the present time. Nevertheless, it is extremely important to present objective growth charts for infant twins using the best available methods. Moreover, there exists much less information on the physical growth of higher-order multiples such as triplets. The growth charts developed in the present study will be more useful than those of the general population to estimate the growth of higher-order multiples.

In conclusion, the present study reveals the physical growth characteristics of twins in Japan and shows the need for early-childhood growth charts for twins, though the present data also suggest that such charts are not necessary for twins after 6 years of age. The present charts are the first objective ones for twins in Japan, and will be especially useful for child and maternal health.
